# Incidence and outcomes of uterine rupture among women with prior caesarean section: WHO Multicountry Survey on Maternal and Newborn Health

**DOI:** 10.1038/srep44093

**Published:** 2017-03-10

**Authors:** Kenichiro Motomura, Togoobaatar Ganchimeg, Chie Nagata, Erika Ota, Joshua P. Vogel, Ana Pilar Betran, Maria Regina Torloni, Kapila Jayaratne, Seung Chik Jwa, Suneeta Mittal, Zenaida Dy Recidoro, Kenji Matsumoto, Mikiya Fujieda, Idi Nafiou, Khalid Yunis, Zahida Qureshi, Joao Paulo Souza, Rintaro Mori

**Affiliations:** 1 Department of Allergy and Clinical Immunology, National Research Institute for Child Health and Development, Tokyo, Japan; 2 Department of Global Health Nursing, Faculty of Medicine, University of Tsukuba, Tsukuba, Japan; 3 Department of Education for Clinical Research, National Center for Child Health and Development, Tokyo, Japan; 4 Global Health Nursing, Graduate School of Nursing Science, St. Luke’s International University, Tokyo, Japan; 5 UNDP/UNFPA/UNICEF/WHO/World Bank Special Programme of Research, Development and Research Training in Human Reproduction (HRP), Department of Reproductive Health and Research, World Health Organization, Geneva, Switzerland; 6 Department of Internal Medicine, Evidence Based Healthcare Post-Graduate Programme, São Paulo Federal University, São Paulo, Brazil; 7 Maternal & Child Morbidity & Mortality Unit, Family Health Bureau, Ministry of Health, Colombo, Sri Lanka; 8 Sora no Mori Clinic, Okinawa, Japan; 9 Obstetrics & Gynecology, Fortis Memorial Research Institute, Gurgaon, India; 10 National Center for Disease Prevention and Control, Department of Health, Manila, Philippines; 11 Department of Pediatrics, Kochi Medical School, Kochi University, Kochi, Japan; 12 Université Abdou Moumouni de Niamey, Niamey, Niger; 13 American University of Beirut, Beirut, Lebanon; 14 Obstetrics and Gynaecology, University of Nairobi School of Medicine, Nairobi, Kenya; 15 Department of Social Medicine, Ribeirão Preto Medical School, University of São Paulo, São Paulo, Brazil; 16 Department of Health Policy, National Center for Child Health and Development, Tokyo, Japan

## Abstract

Caesarean section (CS) is increasing globally, and women with prior CS are at higher risk of uterine rupture in subsequent pregnancies. However, little is known about the incidence, risk factors, and outcomes of uterine rupture in women with prior CS, especially in developing countries. To investigate this, we conducted a secondary analysis of the World Health Organization Multicountry Survey on Maternal and Newborn Health, which included data on delivery from 359 facilities in 29 countries. The incidence of uterine rupture among women with at least one prior CS was 0.5% (170/37,366), ranging from 0.2% in high-Human Development Index (HDI) countries to 1.0% in low-HDI countries. Factors significantly associated with uterine rupture included giving birth in medium- or low-HDI countries (adjusted odds ratio [AOR] 2.0 and 3.88, respectively), lower maternal educational level (≤6 years) (AOR 1.71), spontaneous onset of labour (AOR 1.62), and gestational age at birth <37 weeks (AOR 3.52). Women with uterine rupture had significantly higher risk of maternal death (AOR 4.45) and perinatal death (AOR 33.34). Women with prior CS, especially in resource-limited settings, are facing higher risk of uterine rupture and subsequent adverse outcomes. Further studies are needed for prevention/management strategies in these settings.

Use of caesarean section (CS) deliveries has been steadily increasing, from 6.7% in 1990 to 19.1% in 2014 globally^1^,^2^. Consequently, the number of deliveries by mothers with prior CS is also on the rise^1^.

Women with prior CS are at higher risk of uterine rupture. The reported incidence of uterine rupture among women with prior CS ranged from 0.22% to 0.5% in some developed countries^3^,^4^,^5^,^6^. The risk factors for uterine rupture in women with a history of CS include prior classical incision, labour induction or argumentation, macrosomia, increasing maternal age, post-term delivery, short maternal stature, no prior vaginal delivery, and prior periviable CS^4^,^7^,^8^,^9^,^10^,^11^. Uterine rupture poses considerable risk of adverse maternal and perinatal outcomes. The prevalence of maternal and perinatal complications, such as severe post-hemorrhagic anemia, major puerperal infection, bladder injury, hysterectomy, and perinatal mortality, are significantly higher in women with uterine rupture than women without uterine rupture^4^,^10^,^12^,^13^.

A World Health Organization (WHO) systematic review to determine the prevalence of uterine rupture worldwide identified uterine rupture as a serious obstetric complication being more prevalent and with more serious consequences in developing countries than in developed countries^14^. In developing countries, uterine rupture has been reportedly associated with obstructed labour, grand multiparity, injudicious obstetric interventions/manipulations, lack of antenatal care, unbooked status, poor access to emergency obstetric care, and low socioeconomic status rather than prior CS^15^,^16^,^17^,^18^. However, uterine rupture after prior CS is becoming more common as the availability of CS increases in these settings^18^. According to a literature review on uterine rupture in developing countries, the proportion of women with prior CS or uterine scar among women who had uterine rupture was up to 64%^18^. A study in India reported that the incidence of uterine rupture among women with prior CS was 1.69%^19^. Nevertheless, there are few studies about the incidence, risk factors, and outcomes of uterine rupture among women with prior CS from these settings.

Typically, uterine rupture occurs suddenly and requires immediate critical emergency care for mothers, fetuses, or neonates. The strategies for prevention and management, as well as the quality of affordable care for women at risk of or experiencing uterine rupture, are likely to vary across settings depending on their diagnostic capacity, availability of obstetric interventions, and human and facility resources. Therefore, the findings in developed countries may not be generalizable to low-resource countries and settings. The aim of this analysis was to describe the incidence, risk factors, and maternal and perinatal outcomes of uterine rupture among women with prior CS using data from the WHO Multicountry Survey on Maternal and Newborn Health (WHOMCS), which was conducted in facilities in 29 countries worldwide from 2010 to 2011.

## Methods

### Study design and data collection

We conducted secondary data analysis of the WHOMCS. The original study employed a multistage cluster sampling method to select 359 health facilities in two randomly selected provinces and capital cities of 29 countries in Africa, Asia, Latin America, and the Middle East. The study methods and implementation have been published in detail elsewhere^20^,^21^. In participating facilities, all women undergoing childbirth, as well as women with severe maternal morbidity and/or who died (regardless of the gestational age of the child or the delivery status), were recruited during the study period between May 1, 2010 and December 31, 2011. Trained medical staff at each health facility collected individual data from the medical records, including demographic and obstetric characteristics, and medical conditions during pregnancy, birth outcomes, complications, and received interventions. Characteristics of each health facility were obtained through an institutional survey form completed by the head of the facility or the obstetrics department. Data were collected for two months in facilities with more than 6,000 deliveries per year, and for three months in facilities with less than 6,000 deliveries per year.

The technical content of the research protocol was reviewed and approved by the Research Project Review Panel at the UNDP/UNFPA/UNICEF/WHO/World Bank Special Programme of Research, Development and Research Training in Human Reproduction. The WHOMCS was approved by the WHO Ethical Review Committee and the relevant ethics clearance bodies in participating countries and facilities. The study was conducted in accordance with the principles of Declaration of Helsinki. Written consent from individual women was not needed because there was no personal identification or contact between the data collectors and individual women, and all data were anonymous.

### Study population

The target population for this study was women with at least one prior CS, with a singleton pregnancy, who gave birth in the participating facilities at more than 22 weeks’ gestation or to an infant weighing at least 500 g. We excluded multiple births and women with missing information on uterine rupture, gestational age, or birth weight.

### Variables and definitions

The main variable of interest was uterine rupture in the current pregnancy, which was recorded as a “yes/no” answer in the dataset. Adverse outcomes in this analysis were maternal and perinatal outcomes, which have been used in previous secondary analyses of this dataset^21^,^22^,^23^. Maternal near miss and maternal death were considered as adverse maternal outcomes, whereas fresh stillbirth and intra-hospital early neonatal mortality (IHENM) were considered as adverse perinatal outcomes.

Severe maternal outcomes were defined as maternal death or maternal near-miss cases that occurred from pregnancy through to the eighth day postpartum. Maternal near miss refers to women who presented with a life-threatening condition (i.e., failure or dysfunction of any of the vital organ systems, such as circulatory, respiratory, cardiac, renal, hepatic, central nervous, metabolic, and haematological), as defined by the WHO criteria, and nearly died but survived pregnancy, childbirth, or a pregnancy termination^23^. IHENM was the death of a live-born neonate within the first week of life or before hospital discharge. Perinatal death included fresh stillbirth and IHENM.

Individual, health facility, and country characteristics were considered as covariates in the analysis. Women’s characteristics included maternal age (<20, 20–35, or >35 years), maternal educational level (≤6, 7–12, or >12 years), marital status (single or married/cohabiting), number of prior CS (1, 2, or ≥3), gestational age (<37, 37–41, or ≥42 weeks), birth weight (<2,500, 2,500–3,999, or ≥4,000 grams), onset of labour (spontaneous, induced, or pre-labour CS), fetal presentation (cephalic or non-cephalic), and final mode of delivery (vaginal, caesarean section, or laparotomy caused by uterine rupture). Health facility capacity index was used as a proxy for the institution’s capacity for provision of essential and comprehensive obstetric care and additional services and was calculated as the total score of available services, with further categorization into low, medium, and high^22^. The Human Development Index (HDI) was used for country characteristics and is based on the 2012 rankings (very high/high, medium, and low)^24^.

### Analysis and statistical methods

We described the number and proportion of women with prior CS among all deliveries and uterine rupture in women who had prior CS in the WHOMCS. Thereafter, we described the characteristics of women with prior CS who did not have uterine rupture and had uterine rupture in the current pregnancy. Crude odds ratios (ORs) and adjusted odds ratios (AORs) were calculated to assess the risk factors of uterine rupture in women with prior CS. In this analysis, we used slightly different categories for birth weight (<2,500 or ≥2, 500 grams) and gestational age (<37 or ≥37 weeks) and excluded final mode of delivery, as it included consequence of uterine rupture (i.e., laparotomy caused by uterine rupture).

Finally, we calculated the incidence of maternal and perinatal adverse outcomes among women with prior CS, with and without uterine rupture. Crude ORs and AORs of adverse outcomes were calculated for women who had uterine rupture compared to women who did not have uterine rupture among deliveries with prior CS.

Crude ORs were adjusted for the hierarchical study design (i.e., health facilities as sampling units and countries as strata). For all models, we fitted multilevel logistic regression models with random effects of health facilities. In models of adverse maternal outcomes, adjustments were made for maternal age, maternal educational level, marital status, number of prior CS, health facility capacity, and countries’ HDI group. Gestational age and birth weight were additionally adjusted in models of perinatal outcomes. We did not adjust for mode of delivery because it is in the causal pathway between uterine rupture and adverse outcomes.

We reported all AORs with corresponding 95% confidence intervals (CIs). Missing values were excluded from all logistic regression models. Statistical analysis was conducted using Stata/MP version 13.0 (StataCorp LP, College Station, Texas, USA).

## Results

**Figure 1:**
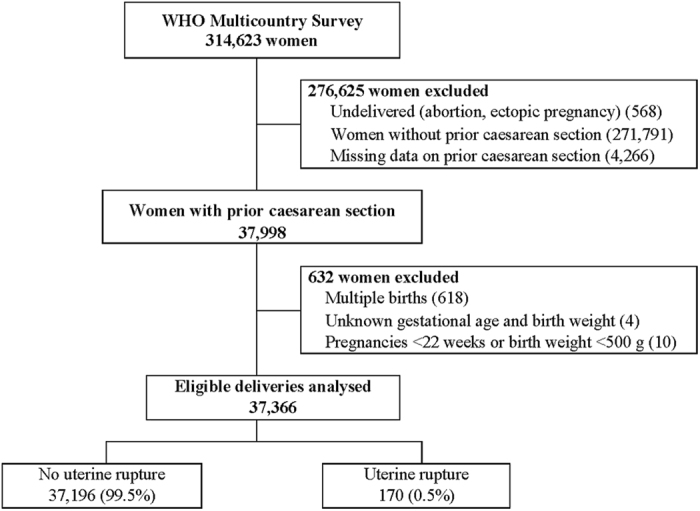
Study sample selection flow chart.

During the study period, the WHOMCS collected data on 314,623 pregnant women in facilities in 29 countries, including 20 medium- and low-HDI countries. Data on 37,366 women (11.8%) were included for this secondary analysis, after excluding women without prior CS (271,791), non-delivered abortion/ectopic pregnancies (568), women with missing information on prior CS (4,266), multiple births (618), unknown gestational age and birth weight (4), and pregnancies <22 weeks or birth weight <500 g (10). Among women included in this analysis, the incidence of uterine rupture was 0.5% (170/37,366) (Fig. 1).

The number of women with prior CS and uterine rupture by HDI is shown in Table 1. The incidence of uterine rupture among women with prior CS was 0.3% in the very high-HDI group, 0.2% in the high-HDI group, 0.4% in the medium-HDI group, and 1.0% in the low-HDI group. Further stratification by country and by number of prior CS is shown in Supplementary Tables S1 and S2. The proportion of women with prior CS ranged from 2.7% in Afghanistan to 24.6% in Mexico.

**Figure 2:**
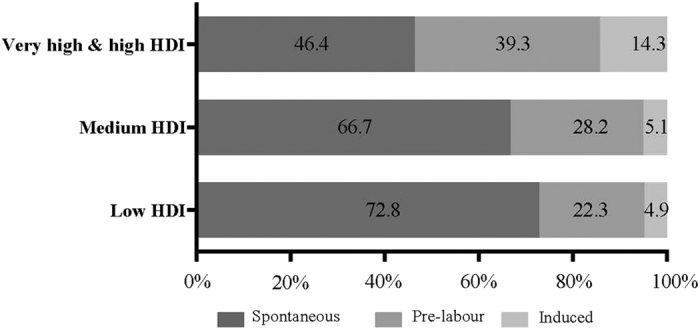
Onset of labour in women with prior caesarean section who had uterine rupture in the current pregnancy by Human Development Index (HDI) group.

Table 2 shows the characteristics of women with prior CS with and without uterine rupture in the current pregnancy. Overall, most of the women with prior CS were aged 20–35 years, married, had one prior CS, and delivered a fetus in cephalic presentation by CS between 37 and 41 weeks of gestation in the current pregnancy. Table 3 presents the relationship between potential risk factors and uterine rupture. In multiple logistic regression analysis, the factors significantly associated with uterine rupture were lower maternal educational level (AOR, 1.71; 95% CI, 1.02–2.87), gestational age at birth less than 37 weeks (AOR, 3.52; 95% CI, 2.14–5.77), spontaneous onset of labour (AOR, 1.62; 95% CI, 1.06–2.46), and delivering in medium- (AOR, 2.00; 95% CI, 1.06–3.77) and low-HDI (AOR, 3.88; 95% CI, 2.05–7.33) countries. The onset of labour among women with prior CS and uterine rupture in the current pregnancy by HDI group is shown in Fig. 2. The proportion of women with spontaneous onset of labour was higher in the low-HDI group (72.8%) than in the very high-/high-HDI group (46.4%), while the proportion of pre-labour CS and induced labour was higher in the very high-/high-HDI groups (39.3% and 14.3%, respectively) than in the low-HDI group (22.3% and 4.9%, respectively). Onset of labour and final mode of delivery are reported by country in Supplementary Table S3.

Comparison of adverse maternal and perinatal outcomes in women with and without uterine rupture among all women with prior CS is shown in Table 4. Overall, severe maternal outcomes occurred in 382 women (1.0%), and there were 792 perinatal deaths (2.1%) in women with prior CS in the study period. Multiple logistic regression analysis found that uterine rupture was associated with significant increases in all pre-specified adverse outcomes: maternal near miss (AOR, 45.25; 95% CI, 26.45–77.42), maternal mortality (AOR, 4.45; 95% CI, 1.15–17.26), severe maternal outcomes (AOR, 40.22; 95% CI, 24.01–67.36), fresh stillbirth (AOR, 59.56; 95% CI, 38.29–92.64), IHENM (AOR, 8.95; 95% CI, 3.72–21.52) and perinatal death (AOR, 33.34; 95% CI, 21.59–51.51). The number and incidence of adverse outcomes among women with prior CS by country are shown in Supplementary Table S4. To investigate whether adverse outcomes of uterine rupture among women with prior CS varied across HDI groups, we further stratified adverse outcomes of uterine rupture by HDI group, and the results are shown in Supplementary Table S5.

Supplementary Table S6 shows the number of women with pregnancy complications and the number and proportion of women who were referred from other hospitals. Supplementary Table S7 shows the proportion of women with preterm deliveries (<37 weeks) in women with prior caesarean section who had uterine rupture in the current pregnancy, by onset of labour and HDI group.

## Discussion

The incidence of uterine rupture in women with prior CS varied across countries, ranging from 0.1% to 2.5% in our sample of 359 facilities in 29 countries worldwide. After adjusting for country-, facility- and individual-level effects, the risk of uterine rupture in women with prior CS was associated with giving birth in medium- or low-HDI countries, spontaneous onset of labour, lower maternal educational level, and gestational age at birth less than 37 weeks. Women with uterine rupture had a significantly higher risk of adverse maternal and perinatal outcomes.

Previous studies reported the incidences of uterine rupture in women with prior CS from 0.22% to 1.69%^3^,^4^,^5^,^6^,^14^,^19^ and these were similar to the results of this study, with an overall rate of 0.5%. The incidence of uterine rupture was highest in low-HDI countries (1.0%), and the multivariate analysis identified giving birth in low-HDI countries as a factor associated with uterine rupture. However, it should be noted that the numbers of observed uterine ruptures were very small in some countries (e.g. 14 countries had 3 or less cases of uterine rupture), and this may affect the reliability of the calculated incidence rates. In addition, the WHOMCS includes both women booked at the facility and women referred/transferred to the facility (Supplementary Table S6). Hence, the incidence rates of uterine rupture are not necessarily comparable across the participating facilities/countries, as they are likely to be affected by the proportion of transferred/referral cases.

In this analysis, women with spontaneous onset of labour had a higher incidence of uterine rupture compared to women who had a pre-labour CS. Although previous studies have shown an association between uterine rupture and labour induction^4^,^6^,^10^,^25^, our analysis did not show a significant increase in risk of uterine rupture among women with induced labour. This may be due to the relatively small number of women who had induced labour in our dataset. Notably, the proportion of women with uterine rupture who had entered labour spontaneously in low-HDI countries (72.8%) was higher than that in very-high/high-HDI countries (46.4%) (Fig. 2). Unfortunately, we did not collect data regarding whether these women were supposed to have a trial of labour after caesarean (TOLAC) or went into labour unexpectedly before their planned CS. The proportions of women who had a vaginal birth after CS (VBAC) varied across countries (Supplementary Table S3). These results may help us understand how women with prior CS are managed and give birth, especially in low-HDI countries where the availability of relevant data is limited.

We found that lower maternal educational level was an independent risk factor for uterine rupture among women with prior CS; in other words, women with a lower educational level are an at-risk sub-population for uterine rupture, likely due to other underlying associations such as social and health inequities. Although we were not able to further investigate the reasons why these women were at higher risk, women with a lower educational level are likely subjected to multiple barriers in accessing and utilizing care, such as those described by the three-delay model suggested by Thaddeus^26^ (i.e., delay in decision to seek care, delay in access to health care, and delay in receiving appropriate and timely interventions).

We observed a significant relationship between gestational age at birth less than 37 weeks and uterine rupture in women with prior CS (AOR 3.52; 95% CI, 2.14–5.77). However, this result should be interpreted carefully because of possible reverse causality; delivery before 37 weeks is likely to be the result of uterine rupture. Higher gestational age is usually reported to be a risk factor for uterine rupture^6^,^10^, but we could not conduct this comparison due to the limited number of deliveries at or after 42 weeks. Notably, 50% (14/28) of uterine rupture occurred before 37 weeks of gestation in very high-/high-HDI countries, and 40% (18/45) of uterine rupture that occurred before labour was observed before 37 weeks of gestation (Supplementary Table S7).

We showed higher risks of severe maternal outcomes (40 times higher) and perinatal death (33 times higher) in women with uterine rupture, compared to women without rupture, which were consistent with prior literature^4^,^6^,^12^. Again, these results suggest that uterine rupture poses a significant risk for the mothers and fetuses/neonates. We compared adverse maternal outcomes among the three HDI groups (very high/high, medium, and low), and middle-HDI countries had the lowest incidence of severe maternal outcomes among women with uterine rupture, although the number of observed events was too small to draw a reliable conclusion. Perinatal death doubled in low-HDI countries compared with that in very high-/high-HDI countries.

This study has several strengths. The WHOMCS was conducted in 29 countries in Africa, Asia, Latin America, and the Middle East, including 20 countries from medium- and low-HDI countries that might have higher incidences of uterine rupture than developed countries. Previous studies on uterine rupture in developing countries revealed only limited information because of small sample sizes. This is the largest multicountry analysis of the incidence, risk factors, and adverse outcomes of uterine rupture in women with prior CS.

Nonetheless, there are several limitations in this study. First, the WHOMCS data collection form did not differentiate between complete and partial uterine rupture and uterine dehiscence. Thus, the diagnosis of uterine rupture might have been affected by the individual facilities/health care providers’ definition. Second, the absolute numbers of uterine ruptures and adverse outcomes that occurred were very small, especially when data were stratified by HDI group and/or country. Therefore, these findings should be interpreted with caution and may not represent the true incidence of uterine rupture and its adverse outcomes. Third, we could not obtain information on several variables known to contribute to uterine rupture, such as TOLAC^27^, type of uterine incision in the previous CS^7^, interval between prior CS and current delivery^25^, and methods of augmentation or induction of labour^28^. Given that TOLAC is likely to affect the results of this study, we adjusted and analyzed the data by onset of labour to compensate for this. Fourth, gestational age was determined based on the best obstetric estimate and local protocols and may have varied in accuracy across facilities/health providers.

In conclusion, based on a large multicountry dataset from 29 countries including developing countries, we obtained the incidence of uterine rupture in women with prior CS ranging from 0.2% in high-HDI countries to 1.0% in low-HDI countries. Identified risk factors included giving birth in medium- or low-HDI countries, spontaneous onset of labour, and lower maternal educational level. Women with uterine rupture had a significantly higher risk of adverse maternal and perinatal outcomes. There are still uncertainties regarding how women with prior CS are managed, especially in low-resource settings. Further studies are needed to identify optimal strategies to prevent/manage uterine rupture in these settings.

## Additional Information

**How to cite this article:** Motomura, K. *et al*. Incidence and outcomes of uterine rupture among women with prior caesarean section: WHO Multicountry Survey on Maternal and Newborn Health. *Sci. Rep.* **7**, 44093; doi: 10.1038/srep44093 (2017).

**Publisher's note:** Springer Nature remains neutral with regard to jurisdictional claims in published maps and institutional affiliations.

## Supplementary Material

Supplementary Information

## Figures and Tables

**Table 1:** Total number of deliveries, number and proportion of women with prior caesarean section among total deliveries, and number and proportion of women with uterine rupture among women with prior caesarean section, by Human Development Index.

HDI	Total deliveries	Women with prior CS			
		Total		Uterine rupture	
		n	%	n	%
Very high-HDI countries	17,294	2,843	16.4	8	0.3
High-HDI countries	68,066	13,125	19.3	20	0.2
Medium-HDI countries	104,206	11,280	10.8	39	0.4
Low-HDI countries	125,030	10,118	8.1	103	1.0
All countries	314,623	37,366	11.9	170	0.5

CS, caesarean section; HDI, Human Development Index.

**Table 2:** Characteristics of women with prior caesarean section, with or without uterine rupture.

Characteristics	Prior CS and no uterine rupture n = 37,196		Prior CS and uterine rupture n = 170		
	n	%	n	%	
Maternal age, years					
<20	1,033	2.8	5	2.9	
20–35	29,127	78.3	133	78.2	
>35	6,960	18.7	29	17.1	
Missing	76	0.2	3	1.8	
Marital status					
Single	2,649	7.1	11	6.5	
Married	34,266	92.1	155	91.2	
Missing	281	0.8	4	2.3	
Education, years					
≤6	5,481	14.7	54	31.8	
7–9	7,034	18.9	28	16.5	
10–12	11,750	31.6	31	18.2	
>12	10,348	27.8	32	18.8	
Missing	2,596	7.0	25	14.7	
Number of previous CS					
1	28,637	77.0	126	74.1	
2	6,972	18.7	35	20.6	
≥3	1,587	4.3	9	5.3	
Gestational age, weeks					
<37	3,077	8.3	48	28.2	
37–41	33,632	90.4	118	69.4	
≥42	399	1.1	2	1.4	
Missing	88	0.2	2	1.4	
Birth weight, grams					
<2500	3,794	10.2	44	25.9	
2500–3999	31,615	85.0	117	68.8	
≥4000	1,739	4.7	6	3.5	
Missing	48	0.1	3	1.8	
Fetal presentation					
Cephalic	34,762	93.5	150	88.2	
Non-cephalic	2,434	6.6	20	11.8	
Onset of labour					
Spontaneous	18,944	51.0	114	67.1	
Induced	2,129	5.7	11	6.5	
Pre-labour	16,007	43.0	45	26.4	
Missing	116	0.3	0	0.0	
Final mode of delivery					
Vaginal	6,877	18.5	5	2.9	
Caesarean section	30,319	81.4	127	74.7	
Laparotomy caused by uterine rupture	0	0.0	38	22.4	
Facility capacity					
High	8,981	24.1	37	21.8	
Medium	16,286	43.8	59	34.7	
Low	6,888	18.5	50	29.4	
Missing	5,041	13.6	24	14.1	
Country HDI					
Very high & high	15,940	42.9	28	16.5	
Medium	11,241	30.2	39	22.9	
Low	10,026	26.9	103	60.6	

CS, caesarean section; HDI, Human Development Index.

**Table 3:** Potential risk factors of uterine rupture in women with prior caesarean section.

		Crude OR (95% CI)		AOR (95% CI)	
Maternal age, years	<20	1.03	(0.31–3.24)	0.97	(0.29–3.16)
	20–35	1		1	
	>35	0.85	(0.50–1.45)	0.90	(0.55–1.49)
Marital status	Married/cohabited	1		1	
	Single	1.15		1.00	(0.45–2.22)
Education, years	≤6	2.97	(1.85–4.77)^***^	1.71	(1.02–2.87)^*^
	7–9	1.31	(0.73–2.35)	1.06	(0.61–1.87)
	10–12	0.70	(0.41–1.19)	0.64	(0.36–1.12)
	>12	1		1	
Number of previous CS	1	1		1	
	2	1.09	(0.62–1.92)	0.96	(0.60–1.55)
	≥3	1.63	(0.79–3.39)	1.36	(0.64–2.88)
Gestational age, weeks	<37	4.70	(3.11–7.11)^***^	3.52	(2.14–5.77)^***^
	≥37	1		1	
Birth weight, grams	<2,500	3.32	(2.09–5.28)^***^	1.42	(0.85–2.36)
	≥2,500	1		1	
Onset of labour	Spontaneous	1.94	(1.12–3.32)^*^	1.62	(1.06–2.46)^*^
	Induced	1.91	(0.86–4.24)	1.79	(0.79–4.02)
	Pre-labour	1		1	
Fetal presentation	Cephalic	1		1	
	Non-cephalic	1.98	(1.08–3.64)^*^	1.48	(0.83–2.64)
Facility capacity	High	1		1	
	Medium	1.05	(0.54–2.06)	0.98	(0.55–1.72)
	Low	2.11	(1.08–4.14)^*^	1.21	(0.65–2.26)
Country HDI	Very high & high	1		1	
	Medium	2.28	(1.20–4.36)^*^	2.00	(1.06–3.77)^**^
	Low	5.13	(2.91–9.04)^***^	3.88	(2.05–7.33)^***^

AOR, adjusted odds ratio; CI, confidence interval; CS, caesarean section; HDI, Human Development Index; OR, odds ratio.

Women without uterine rupture served as a comparison group in the regression model.

Crude odds ratios were adjusted for survey design. Adjusted odds ratios were derived from a multilevel logistic regression model, which included all of the variables in Table 3 with random effects of health facility.

^*^p < 0.05 ^**^p < 0.01 ^***^p < 0.001.

**Table 4:** Adverse maternal and perinatal outcomes among women with prior caesarean section who had and did not have uterine rupture in the current pregnancy.

Outcomes	Overall		No uterine rupture		Uterine rupture		Crude OR	(95% CI)	AOR	(95% CI)
	n	%	n	%	n	%				
Number of deliveries	37,366		37,196		170					
Number of live births	36,697		36,611		86					
Adverse maternal outcomes										
Maternal near miss	339	0.9	286	0.8	53	31.2	53.37	(34.70–82.09)^***^	45.25	(26.45–77.42)^***^
Maternal mortality	43	0.1	38	0.1	5	2.9	18.67	(4.15–83.91)^***^	4.45	(1.15–17.26)^***^
Severe maternal outcomes	382	1.0	324	0.9	58	34.1	51.81	(33.23–80.78)^***^	40.22	(24.01–67.36)^***^
Perinatal adverse outcomes										
Fresh stillbirth	437	1.2	364	1.0	73	42.9	91.18	(57.80–143.82) ^***^	59.56	(38.29–92.64)^***^
IHENM^§^	355	1.0	344	0.9	11	12.9	7.18	(3.43–15.04)^***^	8.95	(3.72–21.52)^***^
Perinatal death^§^	792	2.1	708	1.9	84	49.4	54.20	(35.03–83.86)^***^	33.34	(21.59–51.51)^***^

ADR, adjusted odds ratio; CI, confidence interval; IHENM, intra-hospital early neonatal mortality; OR, odds ratio.

Maternal death: death up to the 8^th^ postpartum day or before hospital discharge; Severe maternal outcomes: maternal near miss and/or death; IHENM: death within 7 days after birth or before hospital discharge; Perinatal death: fresh stillbirth and intra-hospital early neonatal mortality.

Percentages in each group except IHENM: numbers of adverse outcomes divided by total deliveries in each group; Percentages in IHENM: numbers of IHENM divided by total livebirths in each group.

Women without uterine rupture served as a comparison group in the regression models. Crude odds ratios were adjusted for survey design. Multilevel logistic regression models with random effects of health facility were adjusted for maternal age, marital status, education, parity, medical conditions during pregnancy, facility capacity, and country Human Development Index level.

^§^Additionally adjusted for gestational age.

^*^p < 0.05 ^**^p < 0.01 ^***^p < 0.001.
