# Supplementary material for: Incidence and outcomes of uterine rupture among women with prior caesarean section: WHO Multicountry Survey on Maternal and Newborn Health
Source: Sci Rep. 2017 Mar 10;7:44093. doi: 10.1038/srep44093 (PMC5345021; doi:10.1038/srep44093)
Supplement: Supplementary Information [file srep44093-s1.pdf]

Incidence and outcomes of uterine rupture among women with prior caesarean section: WHO Multicountry Survey on Maternal and Newborn Health

Kenichiro Motomura[1§], Togoobaatar Ganchimeg[2§], Chie Nagata[3§]*, Erika Ota[4], Joshua P. Vogel[5], Ana Pilar Betran[5], Maria Regina Torloni[6], Kapila Jayaratne[7], Seung Chik Jwa[8], Suneeta Mittal[9], Zenaida Dy Recidoro [10], Kenji Matsumoto[1], Mikiya Fujieda[11], Idi Nafiou[12], Khalid Yunis[13], Zahida Qureshi[14], Joao Paulo Souza[15], and Rintaro Mori[16]
on behalf of the WHO Multicountry Survey on Maternal and Newborn Health Research Network.

[1] Department of Allergy and Clinical Immunology, National Research Institute for Child Health and Development, Tokyo, Japan
[2] Department of Global Health Nursing, Faculty of Medicine, University of Tsukuba, Tsukuba, Japan
[3] Department of Education for Clinical Research, National Center for Child Health and Development, Tokyo, Japan
[4] Global Health Nursing, Graduate School of Nursing Science, St. Luke's International University, Tokyo, Japan
[5] UNDP/UNFPA/UNICEF/WHO/World Bank Special Programme of Research, Development and Research Training in Human Reproduction (HRP), Department of Reproductive Health and Research, World Health Organization, Geneva, Switzerland
[6] Department of Internal Medicine, Evidence Based Healthcare Post-Graduate Programme, São Paulo Federal University, São Paulo, Brazil
[7] Maternal & Child Morbidity & Mortality Unit, Family Health Bureau, Ministry of Health, Colombo, Sri Lanka
[8] Sora no Mori Clinic, Okinawa, Japan
[9] Obstetrics & Gynecology, Fortis Memorial Research Institute, Gurgaon, India
[10] National Center for Disease Prevention and Control, Department of Health, Manila, Philippines
[11] Department of Pediatrics, Kochi Medical School, Kochi University, Kochi, Japan
[12] Université Abdou Moumouni de Niamey, Niamey, Niger
[13] American University of Beirut, Beirut, Lebanon
[14] Obstetrics and Gynaecology, University of Nairobi School of Medicine, Nairobi, Kenya
[15] Department of Social Medicine, Ribeirão Preto Medical School, University of São Paulo, São Paulo, Brazil
[16] Department of Health Policy, National Center for Child Health and Development, Tokyo, Japan

[§]These authors contributed equally.
*Corresponding author

Supplementary table S1. Total number of deliveries, number and proportion of women with prior caesarean section among total deliveries, and number and proportion of women with uterine rupture among women with prior caesarean section, by Human Development Index and country

HDI	Country	Total deliveries	Women with prior CS			
			Total		Uterine rupture	
			n	%	n	%
Very high-HDI countries		17,294	2,843	16.4	8	0.3
	Japan	3,537	275	7.8	3	1.1
	Qatar	3,950	742	18.8	1	0.1
	Argentina	9,807	1,826	18.6	4	0.2
High-HDI countries		68,066	13,125	19.3	20	0.2
	Mexico	13,309	3,269	24.6	5	0.2
	Lebanon	4,044	809	20.0	1	0.1
	Peru	15,285	2,731	17.9	8	0.3
	Brazil	7,058	1,331	18.9	1	0.1
	Ecuador	10,245	2,398	23.4	3	0.1
	Sri Lanka	18,125	2,587	14.3	2	0.1
Medium-HDI countries		104,206	11,280	10.8	39	0.4
	Jordan	1,167	251	21.5	1	0.4
	China	13,277	1,251	9.4	4	0.3
	Thailand	8,973	1,024	11.4	NR	
	Mongolia	7,365	707	9.6	1	0.1
	Paraguay	3,610	767	21.3	3	0.4
	Philippines	10,783	1,155	10.7	4	0.4
	Vietnam	15,437	2,214	14.3	1	0.1
	Nicaragua	6,571	864	13.3	2	0.2
	India	31,318	2,700	8.6	22	0.8
	Cambodia	4,725	176	3.7	1	0.6
	OPT	980	171	17.4	NR	
Low-HDI countries		125,030	10,118	8.1	103	1.0
	Kenya	20,331	2,282	11.2	19	0.8
	Pakistan	13,175	2,588	19.7	15	0.6
	Angola	10,450	581	5.6	11	1.9
	Nigeria	12,841	949	7.5	9	1.0
	Nepal	11,290	606	5.4	3	0.5
	Uganda	10,923	894	8.2	15	1.7
	Afghanistan	26,148	701	2.7	3	0.4
	DRC	8,756	1,069	12.2	17	1.6
	Niger	11,116	448	4.0	11	2.5
All countries		314,623	37,366	11.9	170	0.5

CS, caesarean section; DRC, Democratic Republic of Congo; HDI, Human Development Index; NR, not reported; OPT, Occupied Palestinian Territory.

Supplementary Table S2. Number of women with prior caesarean section and women who had uterine rupture in the current pregnancy stratified by number of prior caesarean sections in participating countries

HDI groups / countries	Women with prior CS					Uterine rupture					
	Total	1 prior CS		≥2 prior CS		Total		1prior CS		≥2 prior CS	
	n	n	%	n	%	n	%	n	%	n	%
Very high HDI	2,843	2,072	72.9	771	27.1	8	0.3	5	0.2	3	0.4
Japan	275	233	84.7	42	15.3	3	1.1	2	0.9	1	2.4
Qatar	742	510	68.7	232	31.3	1	0.1	1	0.2	NR	
Argentina	1,826	1,329	72.8	497	27.2	4	0.2	2	0.2	2	0.4
High HDI	13,125	9,772	74.5	3353	25.5	20	0.2	16	0.2	4	0.1
Mexico	3,269	2,477	75.8	792	24.2	5	0.2	5	0.2	NR	0.0
Lebanon	809	502	62.1	307	37.9	1	0.1	1	0.2	NR	0.0
Peru	2,731	2,104	77.0	627	23.0	8	0.3	5	0.2	3	0.5
Brazil	1,331	1,040	78.1	291	21.9	1	0.1	NR	0.0	1	0.3
Ecuador	2,398	1,559	65.0	839	35.0	3	0.1	3	0.2	NR	0.0
Sri Lanka	2,587	2,090	80.8	497	19.2	2	0.1	2	0.1	NR	0.0
Medium HDI	11280	9531	84.5	1749	15.5	39	0.3	31	0.3	8	0.5
Jordan	251	147	58.6	104	41.4	1	0.4	1	0.7	NR	0.0
China	1,251	1,215	97.1	36	2.9	4	0.3	3	0.2	1	2.8
Thailand	1,024	902	88.1	122	11.9	NR		NR		NR	
Mongolia	707	564	79.8	143	20.2	1	0.1	1	0.2	NR	
OPT	171	104	60.8	67	39.2	NR		NR		NR	
Paraguay	767	530	69.1	237	30.9	3	0.4	2	0.4	1	0.4
Philippines	1,155	844	73.1	311	26.9	4	0.3	4	0.5	NR	
Vietnam	2,214	2,046	92.4	168	7.6	1	0.0	1	0.0	NR	
Nicaragua	864	713	82.5	151	17.5	2	0.2	1	0.1	1	0.7
India	2,700	2,303	85.3	397	14.7	22	0.8	17	0.7	5	1.3
Cambodia	176	163	92.6	13	7.4	1	0.6	1	0.6	NR	
Low HDI	10,118	7,388	73.0	2730	27.0	103	1.0	74	1.0	29	1.1
Kenya	2,282	1,781	78.0	501	22.0	19	0.8	15	0.8	4	0.8
Pakistan	2,588	1,676	64.8	912	35.2	15	0.6	7	0.4	8	0.9
Angola	581	465	80.0	116	20.0	11	1.9	9	1.9	2	1.7
Nigeria	949	702	74.0	247	26.0	9	0.9	8	1.1	1	0.4
Nepal	606	554	91.4	52	8.6	3	0.5	3	0.5	NR	
Uganda	894	611	68.3	283	31.7	15	1.7	11	1.8	4	1.4
Afghanistan	701	536	76.5	165	23.5	3	0.4	2	0.4	1	0.6
DRC	1,069	710	66.4	359	33.6	17	1.6	11	1.5	6	1.7
Niger	448	353	78.8	95	21.2	11	2.5	8	2.3	3	3.2
All regions	37,366	28,763	77.0	8,603	23.0	170	0.5	126	0.4	44	0.5

CS, caesarean section; DRC, Democratic Republic of Congo; HDI, Human Development Index; NR, not reported; OPT, Occupied Palestinian Territory.

Supplementary Table S3. Total number of women with prior caesarean section and proportions of onset of labour and mode of delivery by Human Development Index and country

HDI	Country	Women with prior CS (n)	Onset of labour (%)				Mode of delivery (%)		
			Spontaneous	Induced	Pre-labour	Missing	Vaginal	CS	Laparotomy
Very high-HDI countries		2,843	39.7	3.1	57.1	0.1	23.4	76.5	0.1
	Japan	275	13.5	0.7	85.8	0.0	8.0	92.0	0.0
	Qatar	742	48.3	2.6	49.2	0.0	36.4	63.6	0.0
	Argentina	1,826	40.1	3.7	55.9	0.2	20.5	79.4	0.1
High-HDI countries		13,125	45.9	9.5	44.5	0.1	16.4	83.5	0.1
	Mexico	3,269	59.5	9.8	30.5	0.2	20.1	79.9	0.0
	Lebanon	809	26.2	9.6	64.2	0.0	4.8	95.2	0.0
	Peru	2,731	51.2	1.6	47.3	0.0	22.1	77.7	0.2
	Brazil	1,331	25.7	19.5	54.6	0.2	23.7	76.3	0.0
	Ecuador	2,398	68.6	13.8	17.4	0.3	9.7	90.3	0.0
	Sri Lanka	2,587	18.7	8.4	72.9	0.0	12.0	88.0	0.0
Medium-HDI countries		11,280	52.1	4.2	43.6	0.0	12.3	87.7	0.1
	Jordan	251	23.9	1.2	74.9	0.0	21.5	78.5	0.0
	China	1,251	30.5	1.0	68.4	0.0	5.3	94.6	0.1
	Thailand	1,024	36.5	2.6	60.8	0.0	0.8	99.2	0.0
	Mongolia	707	18.8	1.6	79.5	0.1	3.7	96.3	0.0
	Paraguay	767	60.4	1.2	38.5	0.0	12.4	87.6	0.0
	Philippines	1,155	26.5	0.6	72.9	0.0	14.2	85.7	0.1
	Viet Nam	2,214	86.9	0.8	12.3	0.1	5.9	94.0	0.1
	Nicaragua	864	35.8	23.0	41.2	0.0	5.3	94.6	0.1
	India	2,700	63.7	6.7	29.6	0.0	26.1	73.8	0.1
	Cambodia	176	80.7	0.0	19.3	0.0	26.7	73.3	0.0
	OPT	171	40.9	2.9	56.1	0.0	23.4	76.6	0.0
Low-HDI countries		10,118	59.6	3.3	36.3	0.9	26.5	73.3	0.2
	Kenya	2,282	65.7	4.7	29.7	0.0	25.2	74.7	0.0
	Pakistan	2,588	43.4	2.7	50.4	3.4	18.8	81.0	0.2
	Angola	581	76.9	3.3	19.8	0.0	36.3	63.0	0.7
	Nigeria	949	41.5	2.7	55.7	0.0	21.0	78.9	0.1
	Nepal	606	52.6	1.8	45.5	0.0	15.2	84.5	0.3
	Uganda	894	78.9	1.5	19.6	0.1	29.4	69.8	0.8
	Afghanistan	701	38.7	4.8	56.5	0.0	41.5	58.5	0.0
	DRC	1,069	85.3	3.5	10.9	0.3	34.5	65.5	0.0
	Niger	448	79.5	2.9	17.4	0.2	42.0	57.6	0.5
All countries		37,366	51.0	5.7	43.0	0.3	18.4	81.5	0.1

CS, caesarean section; DRC, Democratic Republic of Congo; HDI, Human Development Index; NR, not reported; OPT, Occupied Palestinian Territory

Supplementary Table S4. Adverse maternal and perinatal outcomes in women with prior CS who had uterine rupture in the current pregnancy in participating countries

HDI groups / countries	All women				No uterine rupture				Uterine rupture			
	SMO		Perinatal death		SMO		Perinatal death		SMO		Perinatal death	
	n	%	n	%	n	%	n	%	n	%	n	%
Very High HDI	30	1.1	21	0.7	27	1.0	19	0.7	3	37.5	2	25.0
Japan	6	2.2	NR		4	1.5	NR		2	66.7	NR	
Qatar	4	0.5	2	0.3	4	0.5	1	0.1	NR		1	100.0
Argentina	20	1.1	19	1.0	19	1.0	18	1.0	1	25.0	1	25.0
High HDI	109	0.8	160	1.2	101	0.8	154	1.2	8	40.0	6	30.0
Mexico	44	1.3	43	1.3	41	1.3	43	1.3	3	60.0	NR	
Lebanon	7	0.9	15	1.9	7	0.9	14	1.7	NR		1	100.0
Peru	30	1.1	39	1.4	27	1.0	36	1.3	3	37.5	3	37.5
Brazil	4	0.3	12	0.9	4	0.3	12	0.9	NR		NR	
Ecuador	4	0.2	29	1.2	4	0.2	27	1.1	NR		2	66.7
Sri Lanka	20	0.8	22	0.9	18	0.7	22	0.9	2	100.0	NR	0.0
Medium HDI	80	0.7	176	1.6	74	0.7	160	1.4	6	15.4	16	41.0
Jordan	2	0.8	4	1.6	2	0.8	4	1.6	NR		NR	
China	8	0.6	4	0.3	8	0.6	4	0.3	NR		NR	
Thailand	9	0.9	2	0.2	9	0.9	2	0.2	NR		NR	
Mongolia	10	1.4	10	1.4	9	1.3	10	1.4	1	100.0	NR	
OPT	1	0.6	3	1.8	1	0.6	3	1.8	NR		NR	
Paraguay	5	0.7	12	1.6	5	0.7	12	1.6	NR		NR	
Philippines	2	0.2	25	2.2	2	0.2	24	2.1	NR		1	25.0
Vietnam	7	0.3	NR		6	0.3	NR		1	100.0	NR	
Nicaragua	6	0.7	8	0.9	5	0.6	8	0.9	1	50.0	NR	
India	29	1.1	103	3.8	26	1.0	88	3.3	3	13.6	15	68.2
Cambodia	1	0.6	5	2.8	1	0.6	5	2.9	NR		NR	
Low HDI	163	1.6	435	4.3	122	1.2	375	3.7	41	39.8	60	58.3
Kenya	14	0.6	95	4.2	10	0.4	80	3.5	4	21.1	15	78.9
Pakistan	28	1.1	108	4.2	22	0.9	94	3.7	6	40.0	14	93.3
Angola	11	1.9	49	8.4	5	0.9	43	7.5	6	54.5	6	54.5
Nigeria	24	2.5	22	2.3	18	1.9	19	2.0	6	66.7	3	33.3
Nepal	2	0.3	21	3.5	1	0.2	19	3.2	1	33.3	2	66.7
Uganda	16	1.8	36	4.0	11	1.3	29	3.3	5	33.3	7	46.7
Afghanistan	15	2.1	24	3.4	13	1.9	23	3.3	2	66.7	1	33.3
DRC	19	1.8	49	4.6	11	1.0	39	3.7	8	47.1	10	58.8
Niger	34	7.6	31	6.9	31	7.1	29	6.6	3	27.3	2	18.2
All regions	382	1.0	792	2.1	324	0.9	708	1.9	58	34.1	84	49.4

CS, caesarean section; DRC, Democratic Republic of Congo; HDI, Human Development Index; NR, not reported; OPT, Occupied Palestinian Territory; SMO, Severe maternal outcomes, maternal near miss and /or death; Perinatal death, fresh stillbirth and intra-hospital early neonatal mortality.

Supplementary Table S5. Incidence of maternal and perinatal adverse outcomes among women with prior caesarean section who had uterine rupture in the current pregnancy by HDI group

Adverse outcomes	HDI groups						
	Very high & high		Medium		Low		
	n	%	n	%	n	%	P value
Number of uterine rupture	28 (0.2)		39 (0.4)		103 (1.0)		
Adverse maternal outcomes							
Maternal near miss (n=53)	10	35.7	6	15.8	37	35.9	0.053
Maternal death* (n=5)	1	3.6	0	0	4	3.9	1.000
Severe maternal outcomes (n=58)	11	39.3	6	15.8	41	39.8	0.019
Adverse perinatal outcomes							
Fresh stillbirth (n=73)	4	14.3	13	34.2	56	54.4	<0.001
IHENM (n=11)	4	14.3	3	7.9	4	3.9	0.110
Perinatal death (n=84)	8	28.6	16	42.1	60	58.3	0.010

HDI, Human Development Index; Maternal death, death up to the 8th postpartum day or before hospital discharge; Severe maternal outcomes, maternal near miss and/or death; IHENM, Intra-hospital early neonatal mortality, death within 7 days after birth or before hospital discharge; Perinatal death, fresh stillbirth and intra-hospital early neonatal mortality. Chi-square test or Fisher's exact test were used as appropriate. *Comparison was made between the very high-/high-HDI groups and the low-HDI group.

Supplementary Table S6. Number of women with pregnancy complications and number and proportion of women who were referred from other hospitals

	All women		No uterine rupture		Uterine rupture		P value
	n/N	%	n/N	%	n/N	%	
Overall	357/3543	10.1	327/3386	9.7	30/157	19.1	<0.001
Very high & high HDI	171/1507	11.4	167/1485	11.3	4/22	18.2	0.303
Medium HDI	32/979	3.3	29/942	3.8	3/37	8.1	0.116
Low HDI	154/1057	14.6	131/959	13.7	23/98	23.5	0.009

In the data set, hospital referrals from other hospitals were available only for all women who had any pregnancy-related complications. Fisher’s exact tests or chi-square tests were performed whenever appropriate.

Supplementary Table S7. Proportion of women with preterm deliveries (<37 weeks) in women with prior caesarean section who had uterine rupture in the current pregnancy, by onset of labour and Human Development Index group

	All groups		HDI groups					
			Very high & high		Medium		Low	
	n/N	%	n/N	%	n/N	%	n/N	%
Spontaneous	25/113	22.1	6/13	46.2	7/26	26.9	12/74	16.2
Induced	5/10	50.0	3/4	75.0	1/2	50.0	1/4	25.0
No labour	18/45	40.0	5/11	45.5	3/11	27.3	10/23	43.5
All	48/168	28.6	14/28	50.0	11/39	28.2	23/101	22.8

HDI, Human Development Index
